# Tracking of depressed mood from adolescence into adulthood and the role of peer and parental support: A partial test of the Adolescent Pathway Model

**DOI:** 10.1016/j.ssmph.2023.101440

**Published:** 2023-05-26

**Authors:** Magnus Jørgensen, Otto R.F. Smith, Bente Wold, Tormod Bøe, Ellen Haug

**Affiliations:** a Department of Health Promotion and Development, University of Bergen, Bergen, Norway; b Norwegian Institute of Public Health, Bergen, Norway; c Department of Teacher Education, NLA University College, Bergen, Norway; d Department of Psychosocial Science, University of Bergen, Bergen, Norway; e Regional Centre for Child and Youth Mental Health and Child Welfare, NORCE Norwegian Research Centre, Bergen, Norway

## Abstract

• Adolescent depressed mood predicts adult depressed mood.
• Peer acceptance during adolescence is not associated with adult depressed mood.
• Household income moderates the effect of parental closeness on adult depressed mood.

## 1 Introduction

**Fig. 1:**
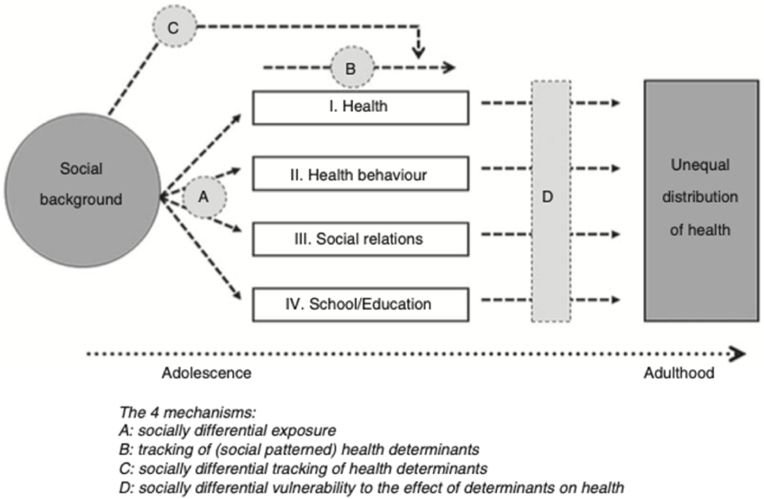
The Adolescent Pathway Model **Note.** Figure showing the Adolescent Pathway Model. From “Pathways and mechanisms in adolescence contribute to adult health inequalities” by Due, P., Krølner, R., Rasmussen, M., Andersen, A., Trab Damsgaard, M., Graham, H., & Holstein, B. E., 2011, *Scandinavian Journal of Public Health*, 39 (6_suppl), 62–78. Copyright 2011 by the Nordic Societies of Public Health.

Depression typically has its onset during adolescence, and like many other psychiatric disorders, continuity is often observed into adulthood (Costello et al., 2003; Fergusson & Woodward, 2002; Wickrama, Conger, Lorenz, & Martin, 2011). For adolescents already at risk due to socioeconomic disadvantage, continuity of depressed mood might be especially pronounced (Wickrama, Conger, Lorenz, & Martin, 2011). This is indicated in that social inequality in depressed mood has been found to persist from early adolescence to mid-adulthood and is in alignment with a large body of knowledge showing an inverse longitudinal association between early life socioeconomic status (SES) and depressive symptomatology later (Elovainio et al., 2012; Stansfeld et al., 2010). According to the Adolescent Pathway Model (APM), adolescence serves as a key developmental period in this respect, with socially patterned pathways stretching from adolescence into adulthood to form long-term health inequalities (Due et al., 2011) (See Fig. 1). As a point of departure in the APM parental SES constitutes the main determinant early in life from which adolescent pathways in health, health behaviors, social relations, and school/education emerge (mechanism A), and it is through these pathways (or health determinants) that parental SES is believed to exert an effect on social inequality of health in adulthood. The health determinants may track over time (mechanism B) and they may do so differently across parental SES groups (mechanism C). The extent to which these health determinants influence social inequality in adult health outcomes may also differ across parental SES-groups, and lower SES groups may, for example, be more vulnerable to their impact than higher SES groups (mechanism D).

## 2 The pervasive influence of household income and parental education

Studies show parents from lower SES face a wider range of stressors due to their financial situation. Furthermore, lower household incomes make it difficult for low SES parents to offer tangible (e.g., money, housing etc.) and emotional support to their offspring (Conger et al., 2010; Prawitz et al., 2012). Similarly, lower parental education is posited to transmit fewer benefits onto adolescents as higher levels of education promotes agency and personal control in individuals. Thus, parents with lower educational attainment don’t seem to be in the same position as their higher SES counterparts to facilitate adolescents’ own development and help them become autonomous individuals who shape their own lives (Mirowsky & Ross, 1998). In short, compared to their more highly educated counterparts, parents with lower educational attainment don’t seem to be as successful in transmitting skills, abilities and character traits to their offspring that protect against depression and other adverse outcomes (Green & Benzeval, 2013). Studies support this by showing that less educated parents don’t seem to develop their offspring’s interpersonal and social skills as much as higher SES parents (Kia et al., 2016; Mohammadi & Zarafshan, 2014). Higher SES parents also offer more social support for their offspring - also when considering the association between social support and SES with different indicators of SES, such as household income, education, and occupation (Arnberg & Melin, 2013; Baheiraei et al., 2012; Huurre et al., 2006).

However, during adolescence, parental emotional support has been postulated to diminish in importance as adolescents become more independent and autonomous and begin to orient themselves more towards peers (while still retaining high levels of instrumental support from parents) (del Valle et al., 2010; Smith et al., 2009; van Eijck et al., 2012; West, 1997). Acceptance from peers has been linked to the development of better social skills that strengthens social support later in life - with lower acceptance from peers being associated with poorer health outcomes and socioeconomic status in adulthood (Almquist & Brännström, 2014; Durlak et al., 2011; Gustafsson et al., 2012; Landstedt et al., 2015; Sakyi et al., 2015). Still, parents constitute an important source of social support for adolescents, for example in the case of adolescent mental health problems as demonstrated in a review of the literature by Gariépy et al. (2016) who found parental support to be most protective against depression in children and adolescents, while support from spouses were more important in adulthood and old age as compared to family and friends. In the same study, support from friends and peers during adolescence was only inconsistently associated with protection against depression (Gariépy et al., 2016).

Given how important social life is for adolescents and the associated sensitive biologically brain development occurring during this life period, it seems plausible that social support during adolescence could have long-lasting implications for adult depressive symptomatology (Allen et al., 2014; Crosnoe & Kirkpatrick Johnson, 2011; Hertzman & Boyce, 2010). However, from the existing literature, it is not evident what role social support from peers and parents during adolescence might play in the long run for depressive outcomes. Adolescence has been suggested to be a sensitive period where exposure to low SES-induced stressors, such as interpersonal struggles, stressful life events etc., could leave lasting marks on individuals’ stress reactivity and alter their depressive trajectories into adulthood (Evans & Kim, 2013; Hertzman & Boyce, 2010; Sisk & Gee, 2022). Social support might be a particularly important pathway to adult depressive symptomatology as it has been closely tied to emotional regulation and stress reactivity (Cassel, 1976; Cobb, 1976; Hostinar & Gunnar, 2015). According to the APM (Due, 2011) the extent to which social support may impact adult depressed mood could differ across SES groups, with lower SES groups more sensitive to the influence of social support than higher SES groups (mechanism D). This has been defined as the social differential vulnerability hypothesis (Vonneilich et al., 2011)*.* Studies support that in families with economic struggles, parents’ preexisting psychological distress and levels of social support for their offspring might be adversely affected by issues tied to limited finances which might have negative ramifications for adolescents’ mental health and continued development into adulthood (Masarik & Conger, 2017; Simons & Steele, 2020). However, the literature is scarce when it comes to studies looking at the effect of parental household income on adult depressive symptomatology across various levels of parental support in a life course perspective.

## 3 The present study

**Fig. 2:**
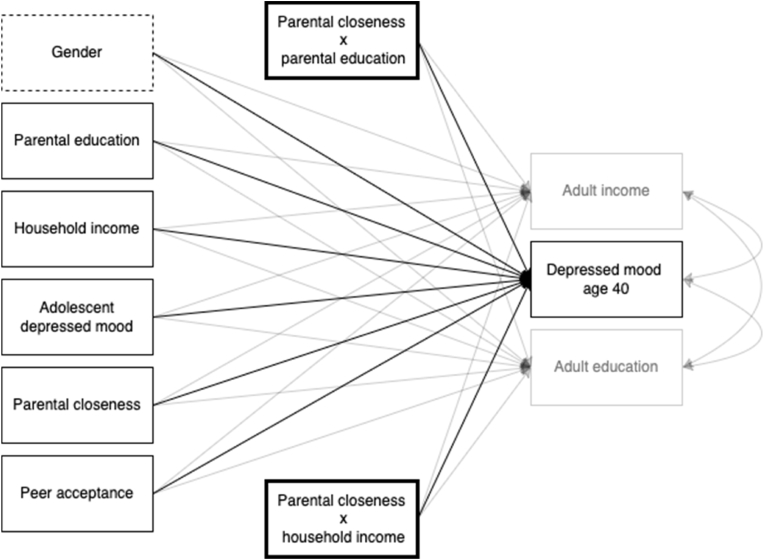
Model 3B **Note.** Model 3B with control variables marked with dotted lines and interaction terms marked with bold lines. Secondary outcomes (adult income and education) are indicated with transparent boxes and lines.

**Fig. 3:**
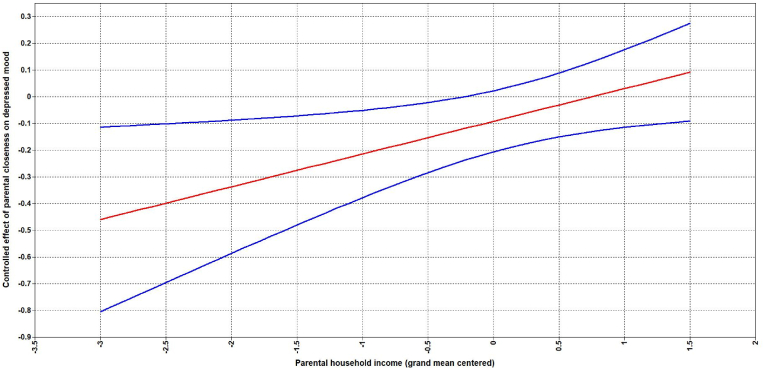
Moderation between household income and parental closeness on adult depressed mood **Note.** Grand mean centered household income on the x-axis. Controlled effect of parental closeness on adult depressed mood on the y-axis. Red line indicating effect estimate and blue lines indicating 95% confidence interval. Intervals not containing zero are considered statistically significant. (For interpretation of the references to colour in this figure legend, the reader is referred to the Web version of this article.)

In the present study, we seek to build upon the existing literature by looking at how social support from peers and parents during adolescence might continue to exert an influence on depressed mood in adulthood as stipulated in a life course perspective - and more specifically in the APM (Due et al., 2011; Kamis & Copeland, 2020). We sought to address this by conducting a partial test of the APM using data from a Norwegian longitudinal study spanning 27-years, covering ages from 13 to 40. More precisely, we investigate how depressed mood tracks over time from early adolescence and onwards with parental closeness and peer acceptance as potential pathways to depressed mood and social inequality in depressed mood in mid-adulthood. That is, we sought to explore (1) whether there is tracking of depressed mood from adolescence into adulthood (mechanism B in Fig. 1), (2) whether this tracking is different across parental SES groups (mechanism C in Fig. 1), and (3) whether the effect of peer acceptance and parental support on depressed mood at age 40 is different across parental SES groups (mechanism D in Fig. 1). For each of these pathways, we also examined to what degree they contributed to explain social inequality in depressed mood at age 40.

## 4 Methods

### 4.1 Sample

Our study used data from the Norwegian Longitudinal Health Behavior Study (NLHB) (n = 1242) with an analytic sample of 1094 Norwegians from Hordaland County in the current study sample (54.3% males). The original NLHB sample was drawn from 22 schools in a random order by following an alphabetical list of schools in the region (using every fifth draw from the list). Respondents were surveyed from age 13 to 40 on a wide range of health and health-related variables in addition to measures of socioeconomic status and living circumstances. In total, participants have been surveyed at ten time points (1990, 1991, 1992, 1993, 1995, 1996, 1998, 2000, 2007 and 2017). In the first three years of the study, respondents completed the questionnaires at school during October and November. Subsequent questionnaires were sent by regular mail during the same months. All surveys were self-administered. In the present study, we used data from 1990, 1992, 1995 and 2017 - based on available data for the study variables and to ensure similar time points were used. No homogenization of scale items was necessary as the scales used in the present study remained intact from 1990 to 2017. Sample characteristics for all study variables are reported in Table 1 and correlation matrix for the study variables is reported in Appendix C.

**Table 1:** Sample characteristics.

	n	Min/Max	M	SD
Household income (1995)	615	1–6	4.29	1.24
Parental education (1995)	968	1–5	2.58	1.18
Adolescent depressed mood (age 13–18)	1061	1–6	2.38	.91
Parental closeness (age 13–18)	1065	1.5–6	4.45	.88
Peer acceptance (age 13–18)	1083	1–6	4.65	.79
Adult income (age 40)	448	1–10	6.44	2.13
Adult educational attainment (age 40)	450	1–5	4.08	1.07
Depressed mood (age 40)	449	1–6	1.63	.83

Note. n = no. of observations, M = mean, Min = Minimum value, Max = Maximum value, SD = Standard Deviation.

The NLHB study was reviewed by the Data Inspectorate of Norway and received a recommendation from the Regional Committee of Medical Research Ethics (REK). Informed written consent has been obtained from participants at every consecutive time point. Information on data collection for NLHB has been reported in further detail elsewhere (Anderssen et al., 2005; Birkeland et al., 2013; Lien et al., 2001). A full attrition analysis for variables included in the present study has been included in Appendix D.

## 5 Instruments

### 5.1 Depressed mood

Depressed mood was measured using a shortened version of the Depressive Tendencies Scale (Alsaker, 1992). Seven items were rated on a 6-point Likert Scale ranging from 1 = does not apply at all to 6 = applies exactly. Items were: ”I think my life is mostly miserable”, ”Sometimes I think my life is not worth living”, ”I am often sad without seeing any reason for it”, ”Sometimes I think everything is so hopeless that I don’t feel like doing anything”, ”I often feel depressed without knowing why”, ”Sometimes I am just so depressed that I feel like staying in bed for the whole day” and ”I don’t think I have anything to look forward to”. The scale has obtained good concurrent validity (r = 0.82) with the CES-D measure of depression in the 1996 wave of the NLHB study (Holsen et al., 2000). McDonald’s omega (ω) indicated good reliability for ages 13, 15, 18 and 40 with estimates of 0.82, 0.87, 0.90. and 0.94. Another study has also established longitudinal partial scalar invariance for the depressive tendencies scale across every time point in the NLHB dataset from age 13 to 40 (Jørgensen et al., 2023).

### 5.2 Measures of perceived social support

Parental closeness was measured using the Parent-Adolescent Scale containing the following items: “My mother and I understand each other well”, “My father and I understand each other well”, “My parents praise and encourage me”, “There is good cohesiveness in my family” and “I enjoy myself when I am together with my parents” (Alsaker et al., 1991). The first three items were measured using a 6-point Likert Scale from 1 = does not apply at all to 6 = applies exactly while the last two items were measured from 1 = seldom or never to 6 = very often. McDonald’s omega (ω) indicated good reliability for ages 13, 15 and 18 with estimates 0.83, 0.83 and 0.85. Peer acceptance was measured using two items on a 6-point Likert Scale ranging from 1 = Very correct to 6 = Incorrect. Items were: “I am doing fine with others my age” and “My peers seem to like me”. Cronbach Alpha for ages 13, 15 and 18 were 0.66, 0.76 and 0.83.

### 5.3 Measures of socioeconomic status

Parents’ report of pretax household income in 1995 was reported in 1996 using one of six categories. Using the 01-01-1995 NOK-EURO exchange rate, this corresponds to “Less than NOK 100.000 (approx. € 11 899)”, “NOK 100–199.000 (approx. € 11 900–23 999)”, “NOK 200–299.000 (approx. € 24 000–34 499)”, “NOK 300–399.000 (approx. € 35 500–46 399)”, “NOK 400–499.000 (approx. € 47 400–59 299)” and “NOK 500.000 or more (approx. € 59 300 or more)”. Parental education was reported in 1996 on a 6-point scale from “0 years of education after elementary school”, “1–2 years of education”, “3 years of education”, “Less than 4 years at university/college”, “More than 4 years at university/college” and “Other”. Missing values, and the last category “other” were replaced with adolescents’ report of parental educational attainment in one of six categories: “Elementary school (6 years, ages 7–12)”, “Upper elementary school (3 years, ages 13–15)”, “Upper secondary school (Vocational)” (ages 16–18), “Upper secondary school (Office/trade) (ages 16–18)”, “Upper secondary school (General studies) (ages 16–18)” and “University/higher education (from age 19)”. This replacement of missing values increased information on parental SES from n = 621 to n = 959. Participants own socioeconomic status as adults (age 40) was also measured as two separate indicators. Adult education was measured using the same categories as reported for parental education while adult income was measured using a 10-point scale which corresponds to the following (01-01-2017 NOK-EURO exchange rate): “Less than NOK 100.000 (approx. € 10 999)”, “NOK 100–199.000 (approx. € 11 000–21 999)”, “NOK 200–299.000 (approx. € 22 000–32 999)”, “NOK 300–399.000 (approx. € 33 000–43 999)”, “NOK 400–499.000 (approx. € 44 000–54 999)”, “NOK 500.000–599.000 (approx. € 55 000–65 999)”, “NOK 600.000–699.000 (approx. € 66 000–76 999)”, “NOK 700.000–799.000 (approx. € 77 000–87 999)”, “NOK 800.000–899.000 (approx. € 88 000–98 999)” and “NOK 900.000 or more (approx. € 99 999 or more)”.

### 5.4 Gender

Gender at age 13 was measured as a binary variable in 1990 with 1 = male and 2 = female.

### 5.5 Statistical analyses

First, we reversed scores for all variables, so that higher scores reflect greater magnitude of the measured construct. Next, we replaced missing values for parental education and computed mean scores for depressed mood and measures of social support. To ensure mean scores and regression estimates were comparable across the survey years (ages 13, 15 and 18), we tested longitudinal measurement invariance for parental closeness and peer acceptance using fit criteria by Chen (2007) (See Appendix B). Our replacement of missing values for parental educational attainment with adolescent reported parental education has been indicated as a fair approach based on an earlier study by Lien et al. (2001). We then proceeded to estimate latent growth factors following guidelines by Duncan and Duncan (2009), but as the growth curves indicated little support for estimating slopes, we settled on intercept (e.g., mean-based) models instead. Next, we tested eight multiple regression models in a hierarchical fashion to better understand how each of our variables (parental SES, adolescent depressed mood, parental closeness, peer acceptance) act as pathways from adolescence to adulthood and how they explain social inequality in adult depressed mood as measured by the covariance between indicators of adult SES and adult depressed mood. In all models, depressed mood at age 40 was used as an outcome measure along with adult education and income. The purpose of including adult education and income was to gauge how socioeconomic inequality in depressed mood was affected by the inclusion of different predictor variables in the models tested. Thus, in the first model (Model 1), we only examined the covariances between the outcome variables, that is, depressed mood along with adult income and education at age 40 as a crude estimate of social inequality in depressed mood at age 40. The next models included additional predictors in the following order: Gender (Model 2A), household income and parental education (Model 2B), adolescent depressed mood (Model 2C – Mechanism B), parental closeness and peer acceptance (Model 2D), interactions between household income and parental education and adolescent depressed mood (Model 3A – Mechanism C), interactions between parental closeness and household income and education (Model 3B – Mechanism D) (See Fig. 2), and finally, interactions between peer acceptance and household income and parental education (Model 3C – Mechanism D). Variables used in interaction terms were grand mean centered and interaction terms were only tested in one model at a time. All analyses were done using Maximum Likelihood Estimation with robust standard errors (MLR) for estimating parameters and Full Information Maximum Likelihood (FIML) for missing values. Preliminary analyses were done in SPSS v. 26 while main analyses were computed in Mplus v. 8.7 (Muthen & Muthen, 2017).

## 6 Results

In the following, results from the regression analyses (Model 2A-3C) are presented - starting with estimates for tracking (mechanism B) and social differential tracking (mechanism C) of depressed mood from adolescence into adulthood, followed by estimates for social differential vulnerability and the effects of parental closeness and peer acceptance during adolescence on adult depressed mood (mechanism D). Finally, the extent to which these different pathways contribute to explain social inequality in adult depressed mood are presented. Standardized estimates for all eight regression models with adult income and education as outcome variables are reported in Appendix A.

### 6.1 Tracking of depressed mood and effects of parental closeness and peer acceptance

Standardized regression estimates are reported in Table 2. We found that depressed mood tracked over time (Model 2C – mechanism B), but the tracking did not vary across parental income and education levels (Model 3A – mechanism C). In contrast, a significant interaction term was found for parental closeness and household income in model 3B - indicating support for Mechanism D (socially differential vulnerability). This interaction effect is depicted in Fig. 3 with plausible values for household income on the x-axis and the effect of parental closeness on depressed mood at age 40 on the y-axis. Fig. 3 indicates that the effect of parental closeness on depressed mood at age 40 becomes more negative for low values of household income and becomes positive for high values of household income. The graph also shows that the effect of parental closeness was only statistically significant for sample values that were below average household income. We found no evidence for mechanism D with regards to peer acceptance.

**Table 2:** Model 2A-3C with depressed mood at age 40 as outcome.

	Model 2A	Model 2B	Model 2C	Model 2D	Model 3A	Model 3B	Model 3C
Gender	0.040 [-0.051, 0.132]	0.035 [-0.057, 0.126]	−0.019 [-0.108, 0.070]	−0.030 [-0.117, 0.058]	−0.033 [-0.121, 0.055]	−0.034 [-0.121, 0.054]	−0.022 [-0.112, 0.067]
Household income	–	−0.043 [-0.193, 0.106]	0.004 [-0.135, 0.143]	0.010 [-0.132, 0.152]l	0.013 [-0.114, 0.139]	−0.012 [-0.126, 0.150]	0.014 [-0.179, 0.010]
Parental education	–	−0.124* [-0.222, -0.026]	−0.088 [-0.185, 0.009]	−0.083 [-0.179, 0.012]	−0.083 [-0.181, 0.015]	−0.088 [-0.179, 0.003]	−0.085 [-0.126, 0.153]
Adolescent depressed mood	–	–	0.306*** [0.212, 0.401]	0.251*** [0.141, 0.362]	0.209* [0.105, 0.313]	0.234*** [0.122, 0.345]	0.241*** [0.131, 0.351]
Parental closeness	–	–	–	−0.099 [-0.217, 0.019]	−0.103 [-0.219, 0.013]	−0.098 [-0.218, 0.023]	−0.104 [-0.223, 0.014]
Peer acceptance	–	–	–	−0.046 [-0.158, 0.067]	−0.044 [-0.157, 0.068]	−0.054 [-0.168, 0.059]	−0.042 [-0.156, 0.071]
Adolescent depressed mood x household income	–	–	–	–	−0.188 [-0.411, 0.035]	–	–
Adolescent depressed mood x parental education	–	–	–	–	0.047 [-0.074, 0.168]	–	–
Parental closeness x household income	–	–	–	–	–	0.164* [0.028, 0.299]	–
Parental closeness x parental education	–	–	–	–	–	−0.009 [-0.123, 0.104]	–
Peer acceptance x household income	–	–	–	–	–	–	0.090 [-0.043, 0.223]
Peer acceptance x parental education	–	–	–	–	–	–	−0.030 [-0.135, 0.074]

Note. Standardized estimates presented with 95% confidence intervals in brackets. Estimates in bold are significantly different from zero (***p < .001, **p < .01,*p < .05).

### 6.2 Covariance between indicators of adult SES and depressed mood

Table 3 shows the inverse relationship between adult SES and depressed mood that was explored in all eight regression models. Model 2B and model 2C were associated with the largest drops in covariance/correlation between depressed mood and parental SES. From Table 2 and appendix A, it can be derived that this was mainly driven by adolescent depressed mood and parental educational level. Together, these two factors explained about 19% of the social inequality in adult depressed mood associated with adult income, and about 24% of the social inequality of adult depressed mood associated with adult education (Model 2C, Table 3) (Calculating percentage change from Model 1 to Model 2C). The interaction effect between parental closeness and parental household income explained an additional 6% of the social inequality in adult depressed mood associated with adult education (Model 3B, Table 3).

**Table 3:** Correlation between adult SES and depressed mood age 40.

	Model 1	Model 2A	Model 2B	Model 2C	Model 2D	Model 3A	Model 3B	Model 3C
Adult depressed mood and adult income	−0.260*** [-0.358, -0.161]	−0.261*** [-0.363, -0.158]	−0.244*** [-0.346, -0.141]	−0.211*** [-0.314, -0.109]	−0.209*** [-0.311, -0.106]	−0.213*** [-0.314, -0.111]	−0.216*** [-0.319, -0.114]	−0.208*** [-0.311, -0.105]
Adult depressed mood and adult education	−0.234*** [-0.340, -0.128]	−0.239*** [-0.345, -0.134]	−0.199*** [-0.304, -0.094]	−0.178** [-0.281, -0.074]	−0.173** [-0.275, -0.070]	−0.171** [-0.275, -0.068]	−0.163** [-0.265, -0.061]	−0.170** [-0.272, -0.067]

Note. Standardized estimates presented with 95% confidence intervals in brackets. Estimates in bold are significantly different from zero (*** *p* < .001, ** *p* < .01, * *p* < .0).

## 7 Discussion

In the present study, we provided a partial test of the Adolescent Pathway Model by investigating how adolescent depressed mood tracks over time from early adolescence and onwards – with indicators of parental SES and peer acceptance and parental closeness from age 13 to 18 as potential pathways to social inequality in depressed mood at age 40. Our main findings show that adolescent depressed mood predicted depressed mood at age 40. This tracking did not vary across household income and parental education levels. Further, we observed an interaction between parental closeness and household income on depressed mood at age 40 - with the effect of parental closeness being statistically significant and negative for values below average household income. The concurrent association between adult SES and depressed mood at age 40 was negative in all regression models – with strongest associations for adult income. Adolescent depressed mood and parental education led to the largest drops in covariance between indicators of adult SES and depressed mood.

### 7.1 Tracking of depressed mood from adolescence into adulthood

The finding that depressed mood tracks from adolescence to adulthood is in alignment with the APM (pathway I and mechanism B). In addition, several empirical studies support this finding by indicating homotypic continuity for depressive symptomatology (Costello et al., 2003). This observed continuity in depressive symptomology might be accounted for by several factors; stressful life events, cognitive biases, personality, and genetics factors have been put forth as possible explanatory mechanisms (Eley & Stevenson, 1999; Gotlib & Hammen, 2014; Kendler et al., 2000). For instance, external factors, such as stressful life events, can precipitate a depressive episode which then leads to recurring depressive episodes that are more autonomous in nature - meaning that they need not be triggered by subsequent major negative life events (Post, 1992), (Lewinsohn et al., 1999; Monroe et al., 1999). Also, onset of depressive symptoms in adolescence have been associated with adverse effects in social, occupational, and academic domains which may accumulate and lead to worsened depressive symptoms in adulthood (Fergusson & Woodward, 2002; Rudolph et al., 2009). For example, one study by Glied and Pine (2002) found that depressed adolescents were more likely to cope with stress by engaging in high-risk behaviors, such as alcohol consumption, smoking and drug use. Other outcomes were also found to be associated with depression, including poor school performance and suicidal ideation (Glied & Pine, 2002). But, as noted by the authors, the study does not allow to establish the exact causal linkage to depression. However, one interpretation could be that risk behaviors lead to reduced life opportunities that further shape depressive trajectories into adulthood. This also fits well with the findings of Wickrama, Conger, Lorenz, & Martin, 2011 which found socioeconomic attainment being linked to disruption in depressive continuity from late adolescence to young adulthood (Wickrama, Conger, Lorenz, & Martin, 2011). This makes sense given that educational and occupational engagements constitute important developmental goals for most emerging adults that could potentially alter depressive trajectories (Wickrama, Conger, Lorenz, & Martin, 2011).

In the present study, we also investigated whether parental income and education resulted in different tracking of depressed mood from adolescence to adulthood (mechanism C in the APM). An earlier study on the same sample used in the present study did reveal stable socioeconomic difference in depressed mood persisting from early adolescence to mid-adulthood (mechanism A in the APM) (Jørgensen et al., 2023). However, the present study did not find this difference in depressed mood to track socioeconomically differently over time. Socially differential tracking of depressive symptomology from younger age to adulthood have only received limited empirical attention previously. One study by Minh et al. (2021) looked at trajectories of depressive symptoms from age 16 to 25 among Canadian and American adolescents with childhood socioeconomic status as a predictor of trajectory membership. Their findings showed that childhood family income predicted an increasing trajectory of depressive symptoms for American, but not Canadian adolescents. They also found parental education at high school level to be associated with a decreasing trajectory compared to a stable low level of depressive symptoms for American adolescents. Another study by Elovainio et al. (2012) on a Finnish sample also did not find socioeconomic status in early adolescence to be predictive of increasing or decreasing depressive trajectories from adolescence to adulthood, however, lower SES was associated with higher depressive symptoms overall across the study period. Both Canada, Norway and Finland offer comprehensive welfare programs. Thus, country-level differences in welfare systems might play a role in shaping development of depressive symptoms over time with differing levels of welfare benefits available for socially disadvantaged pupils (e.g., unemployment compensation, health insurance, housing assistance etc.). Nevertheless, research hasn’t consistently found welfare regimes to buffer against socioeconomic inequality in health (Mackenbach, 2012; McAllister et al., 2018). Finally, it should also be noted that individuals in welfare states do exhibit higher levels of overall mental health as compared to countries with less developed welfare regimes (McAllister et al., 2018).

### 7.2 Long-term effects of parental closeness and peer acceptance

Parental closeness was negatively associated with adult depressed mood for participants reporting below average household income - with the relative impact of parental closeness on adult depressed mood increasing as the reported household income decreased. This indicates a sustained effect of household income for adolescents growing up with different levels of parental closeness. This lends support to mechanism D (socially differential vulnerability) in the APM. This could indicate that household income could protect against some of the adverse effects associated with low parental support such as higher levels of externalizing and internalizing problems, poorer social and academic adjustment and lower quality of life - all of which can affect long-term depressive outcomes through allostatic load (Danese et al., 2009; Luyckx et al., 2014; Lyell et al., 2020; Rueger et al., 2008). These findings are largely in line with a few other studies that have explored the same subject matter, but for different age groups and with shorter time spans (Heritage et al., 2008; Vonneilich et al., 2011). For example, with social support as the moderator of interest, Huurre et al. (2006) examined the association between low parental occupation at age 16 and depression at age 32 with social support from parents, peers, teachers, friends and partners as moderators at ages 16, 22 and 32. Overall, males were more vulnerable to effects of low social support on depression at age 32, but both genders showed moderating effects between low parental occupation and support from same-sex parent at age 16 on depression (age 32). Another study by Vonneilich et al. (2011) looked at Germans aged 45–75 years and whether instrumental and emotional support were associated with self-rated health and depressive symptoms and if these associations were moderated by socioeconomic status. Some evidence for moderation was found, but results differed based on indicators of SES, social support, and gender. Females generally showed more consistent moderation effects by SES - speaking in favor of a social differential vulnerability hypothesis. A study by Heritage et al. (2008) looking at French adults also found that for people with lower income, social isolation had a more negative effect on self-rated health as compared to people with higher incomes. However, some studies have found evidence contrasting the social differential vulnerability hypothesis - showing instead that individuals in higher SES groups are more exposed to the negative effects of low social support on depressive and health-related outcomes (Luo et al., 2021; Melchior et al., 2003; Nguyen et al., 2019; Patterson, 2016). Thus, more research is warranted to figure out whether these inconsistencies present in the literature reflect differences in study designs, instruments, age groups studied, and/or time spans covered.

In the present study, we also looked at long-term effects of peer acceptance, but found no direct effects or moderation effects of SES. This could reflect the changing social relationships of adolescence. That is, in contrast to peers, parents represent more stable and fundamental sources of influences from early childhood and onwards, thus, it is expected that they also have a more long-term impact on adult health compared to more unstable and brief associations with peers (Antonucci et al., 2004). However, the current study somewhat contrast the findings from a longitudinal study in the US that looked at depressive trajectories from age 12 to 32 and found that peer sociality at mid-adolescence predicted depressive trajectories into adulthood – with adolescent peer popularity also predicting the trajectory for women, but not for men (Kamis & Copeland, 2020). However, this study did not control for parental support or moderation by parental SES which could explain differences in findings compared to the present study. Also, our study measured other dimensions of peer relations and did not include concurrent associations between peer acceptance and depressed mood in adolescent years. Furthermore, in the literature more generally, support from peers has been associated with protection against depression during adolescence more inconsistently as compared to parental support (Gariépy et al., 2016). One possible reason for the inconsistent and perhaps short-term influence of peers could be that as adolescents enter adulthood and are faced with new social arenas and developmental tasks, past support from peers during adolescence becomes less relevant (Arnett, 2000).

### 7.3 Pathways to the social gradient in adulthood

A secondary aim of this study was to consider how adolescent health determinants might affect adult social inequality in depressed mood. Across the models tested, a small negative covariance was found between indicators of adult SES and adult depressed mood. Adding measures of social support reduced the covariance very slightly, but parental education along with depressed mood in adolescent years showed the strongest effects on the covariance. The interaction between parental closeness and household income had a comparatively smaller effect. It is possible that measures of social support, such as emotional support from spouses, would account for a larger portion of the association between adult SES and depression at age 40. This is indicated in the meta-analysis by Gariépy et al. (2016) which found spouse support to be more protective against adult depression than other sources of social support. However, given how many other factors that have been linked to social inequalities in health, social support likely only constitutes a small piece of a larger puzzle to better understand the social gradient in depressive outcomes across the life course (Pampel et al., 2010). Still, from the current study, our findings do indicate sustained, long-term effects of parental SES and depressive symptomatology on the social gradient in adulthood.

### 7.4 Limitations

Some limitations of the present study should be noted. Firstly, our data was collected in Norway which is known for its socially egalitarian welfare state with high welfare benefits for all socioeconomic groups - thereby limiting the number of cases with extreme social and economic deprivation. By implication, our sample is rather homogenous with a high baseline level of living conditions and small variance in SES compared to other countries. For later time points, our attrition analysis also indicated some selection effects with higher parental education and household income being associated with higher participation at the last time point. Secondly, our measures of social support did not distinguish between instrumental, informational, appraisal and emotional support. Instead, our items were mostly oriented towards emotional domains – which is important as current research indicates emotional support is the most important predictor of adult depression compared to other types of support (Gariépy et al., 2016). Further, we did not distinguish between maternal and paternal support, because of concerns related to statistical power, however, future studies could benefit from doing so. We also did not measure support from close friends during adolescence, thus, making it more difficult to extrapolate differential effects from different sources of social support. Still, we believe peer acceptance is a highly relevant measure as adolescents, generally, are very concerned with their standing among peers, whether close or not (Brown et al., 1986). Lastly, we should also mention the risk of measurement error in cases where adolescents’ report of parental education has been used as substitute because of missing values.

## 8 Conclusion

The present study indicates partial support for the APM. Depressed mood tracks from ages 13, 15 and 18 to adulthood (though not in socially patterned ways). Social differential vulnerability effects are indicated with household income moderating the effects of parental closeness on depressed mood at age 40 - although this conditional effect was only statistically significant for participants with below average household incomes. Further, our study demonstrated that the social gradient in adult depressed mood was partly explained by adolescent depressed mood and parental education, and to a lesser extent also by the interaction between parental closeness and household income. Our findings add to meta-analyses on the associations between SES, different sources of social support and depressive outcomes, but includes a life course perspective by focusing on social inequality in depressed mood as a long-term outcome. Furthermore, our study suggests that policy initiatives targeting household income inequalities might alleviate some of the adverse effects of low parental closeness on adult depressed mood. Alternatively, inequalities in parental support might also be addressed more directly through interventions that make emotional support more accessible to adolescents by improving parent-adolescent communication in times of distress.

## Financial disclosure

This project is funded by the Norwegian Research Council (Grant number 302225).

## Ethical statement

Hereby, I Magnus Jørgensen, consciously assure that for the manuscript titled “Tracking of depressed mood from adolescence into adulthood and the role of peer and parental support: A partial test of the Adolescent Pathway Model” the following is fulfilled.

1) This material is the authors' own original work, which has not been previously published elsewhere.
2) The paper is not currently being considered for publication elsewhere.
3) The present study used data from the Norwegian Longitudinal Health Behaviour (NLHB) study which has been approved by the Data Inspectorate of Norway and received a recommendation from the Regional Committee of Medical Research Ethics (REK). Informed written consent has been obtained from participants at every consecutive time point in the study.

## CRediT authorship contribution statement

**Magnus Jørgensen:** Formal analysis, Writing – original draft, Writing – review & editing. **Otto R.F. Smith:** Formal analysis, Writing – review & editing, Visualization. **Bente Wold:** Conceptualization, Investigation, Writing – review & editing, Funding acquisition. **Tormod Bøe:** Writing – review & editing, Visualization. **Ellen Haug:** Writing – review & editing, Project administration, Supervision.

## Declaration of competing interest

None.

## Data Availability

Data will be made available on request.
