# Supplementary material for: Tracking of depressed mood from adolescence into adulthood and the role of peer and parental support: A partial test of the Adolescent Pathway Model
Source: SSM Popul Health. 2023 May 26;23:101440. doi: 10.1016/j.ssmph.2023.101440 (PMC10492161; doi:10.1016/j.ssmph.2023.101440)
Supplement: Multimedia component 1 [file mmc1.docx]

**Appendix A. Standardized estimates for regression models with adult education and income at age 40 as outcome variables**

|  | **Model 2a** | **Model 2b** | **Model 2c** | **Model 2d** | **Model 3a** | **Model 3b** | **Model 3c** |
| --- | --- | --- | --- | --- | --- | --- | --- |
| **Adult education** |  |  |  |  |  |  |  |
| Gender | **0.099***  **[0.006, 0.192]** | **-0.349*** [0.027, 0.201]** | **0.133****  **[0.047, 0.220]** | **0.138****  **[0.051, 0.225]** | **0.138****  **[0.051, 0.225]** | **0.139****  **[0.052, 0.226]** | **0.134****  **[0.046, 0.222]** |
| Household income |  | 0.078  [-0.040, 0.197] | 0.063  [-0.057, 0.182] | 0.061  [-0.059, 0.180] | 0.061  [-0.059, 0.180] | 0.056  [-0.063, 0.175] | 0.058  [-0.062, 1.178] |
| Parental education |  | **0.321*****  **[0.229, 0.413]** | **0.308*****  **[0.213, 0.403]** | **0.303*****  **[0.208, 0.398]** | **0.301*****  **[0.204, 0.399]** | **0.308**  **[0.212, 0.403]** | **0.304*****  **[0.209, 0.398]** |
| Adolescent depressed mood |  |  | **-0.108***  **[-0.213, -0.002]** | -0.085  [-0.199, 0.030] | -0.080  [-0.192, 0.033] | -0.076  [-0.190, 0.037] | -0.080  [-0.198, 0.037] |
| Parental closeness |  |  |  | 0.015  [-0.092, 0.123] | 0.016  [-0.092, 0.123] | 0.013  [-0.101, 0.128] | 0.017  [-0.090, 0.125] |
| Peer acceptance |  |  |  | 0.054  [-0.056, 0.164] | 0.054  [-0.055, 0.164] | 0.059  [-0.051, 0.168] | 0.052  [-0.058, 0.163] |
| Adolescent depressed mood x household income |  |  |  |  | 0.027  [-0.094, 0.148] |  |  |
| Adolescent depressed mood x parental education |  |  |  |  | -0.016  [-0.142, 0.110] |  |  |
| Parental closeness x househould income |  |  |  |  |  | -0.086  [-0.230, 0.059] |  |
| Parental closeness x parental education |  |  |  |  |  | 0.020  [-0.089, 0.130] |  |
| Peer acceptance x household income |  |  |  |  |  |  | -0.048  [-0.188, 0.091] |
| Peer acceptance x parental education |  |  |  |  |  |  | 0.021  [-0.098, 0.141] |
|  |  |  |  |  |  |  |  |
| **Adult income** |  |  |  |  |  |  |  |
| Gender | **-0.355*****  **[-0.439, -0.271]** | **0.114***  **[-0.432, -0.265]** | **-0.323*****  **[-0.411, -0.235]** | **-0.321*****  **[-0.408, -0.234]** | **-0.322*****  **[-0.409, -0.235]** | **-0.320*****  **[-0.407, -0.232]** | **-0.321*****  **[-0.408, -0.235]** |
| Household income |  | 0.015  [-0.098, 0.129] | -0.005  [-0.118, 0.109] | -0.013  [-0.125, 0.099] | -0.009  [-0.120, 0.102] | -0.011  [-0.122, 0.101] | -0.014  [-0.125, 0.097] |
| Parental education |  | **0.133****  **[0.035, 0.231]** | **0.115***  **[0.017, 0.212]** | **0.112***  **[0.014, 0.210]** | 0.101  [-0.001, 0.202] | **0.109***  **[0.012, 0.206]** | **-0.112***  **[0.014, 0.209]** |
| Adolescent depressed mood |  |  | **-0.150****  **[-0.243, -0.058]** | **-0.119***  **[-0.224, -0.014]** | **-0.123***  **[-0.231, -0.015]** | **-0.121***  **[-0.224, -0.018]** | **-0.120***  **[-0.225, -0.015]** |
| Parental closeness |  |  |  | -0.005  [-0.110, 0.100] | -0.008  [-0.114, 0.098] | 0.000  [-0.103, 0.104] | -0.005  [-0.110, 0.100] |
| Peer acceptance |  |  |  | 0.084  [-0.017, 0.185] | 0.088  [-0.012, 0.189] | 0.082  [-0.020, 0.184] | 0.083  [-0.018, 0.184] |
| Adolescent depressed mood x household income |  |  |  |  | 0.015  [-0.106, 0.136] |  |  |
| Adolescent depressed mood x parental education |  |  |  |  | -0.063  [-0.184, 0.058] |  |  |
| Parental closeness x househould income |  |  |  |  |  | 0.037  [-0.085, 0.160] |  |
| Parental closeness x parental education |  |  |  |  |  | -0.032  [-0.126, 0.061] |  |
| Peer acceptance x household income |  |  |  |  |  |  | -0.021  [-0.136, 0.094] |
| Peer acceptance x parental education |  |  |  |  |  |  | 0.023  [-0.076, 0.123] |
|  |  |  |  |  |  |  |  |

Note. Standardized estimates presented with 95% confidence intervals in brackets. Estimates in bold are significantly different from zero (*** *p* < .001, ** *p* < .01, * *p* < .05).
