# Supplementary material for: Tracking of depressed mood from adolescence into adulthood and the role of peer and parental support: A partial test of the Adolescent Pathway Model
Source: SSM Popul Health. 2023 May 26;23:101440. doi: 10.1016/j.ssmph.2023.101440 (PMC10492161; doi:10.1016/j.ssmph.2023.101440)
Supplement: Multimedia component 2 [file mmc2.docx]

**Appendix B. Measurement invariance**

|  | *χ*^2^ | *df* | RMSEA [90%CI] | CFI | SRMR | ΔRMSEA | ΔCFI | ΔSRMR |
| --- | --- | --- | --- | --- | --- | --- | --- | --- |
| *The Parent-Adolescent Scale* | |  |  |  |  |  |  |  |
| Configural | 126.166 | 72 | .026  [.019, .034] | .988 | .025 |  |  |  |
| Metric | 146.273 | 80 | .028  [.021, .035] | .985 | .036 | .002 | .003 | .011 |
| Scalar | 301.219 | 90 | .047  [.041, .053] | .951 | .058 | .019 | .034 | .022 |
| Partial Scalar | 199.893 | 88 | .034  [.028, .041] | .974 | .045 | .013 | .023 | .013 |
| *Peer Acceptance* | |  |  |  |  |  |  |  |
| Configural | 38.457 | 6 | .071  [.050, .093] | .970 | .024 |  |  |  |
| Metric | 41.699 | 8 | .062  [.044, .082] | .969 | .027 | .009 | .001 | .003 |
| Scalar | 99.137 | 12 | .082  [.067, .097] | .919 | .060 | .020 | .050 | .033 |

Note. *χ*^2^ = Chi-Square value, df = Degrees of Freedom, RMSEA = Root Mean Square Error of Approximation, CFI = the Comparative Fit Index and SRMR = Standardized Root Mean Square Residual

Partial scalar invariance was established for the Parent-Adolescent Scale (Ages 13, 15 and 18) by freeing intercepts for item four (“There is good cohesiveness in my family”) and five (“I enjoy myself when I am together with my parents”) at age 13. Though CFI was above the cut-off of ≥ 10, neither RMSEA nor SRMR indicated non-invariance from metric to partial scalar. For both depressed mood and the Parent-Adolescent Scale, items were correlated with themselves over time. The scale for peer acceptance only yielded metric invariance (Ages 13, 15 and 18) - indicating that regression estimates, but not means were comparable across time.
