# Supplementary material for: Tracking of depressed mood from adolescence into adulthood and the role of peer and parental support: A partial test of the Adolescent Pathway Model
Source: SSM Popul Health. 2023 May 26;23:101440. doi: 10.1016/j.ssmph.2023.101440 (PMC10492161; doi:10.1016/j.ssmph.2023.101440)
Supplement: Multimedia component 3 [file mmc3.docx]

**Appendix C**

*Correlation matrix for Study Variables*

| Variable | 1 | 2 | 3 | 4 | 5 | 6 | 7 | 8 | | 9 |
| --- | --- | --- | --- | --- | --- | --- | --- | --- | --- | --- |
| 1. Depressed mood age 40 | — |  |  |  |  |  |  | |  |  |
| 2. Adult income | -.260^**^ | — |  |  |  |  |  | |  |  |
| 3. Adult education | -.233^**^ | .225^**^ | — |  |  |  |  | |  |  |
| 4. Gender | .041 | -.357^**^ | .098^*^ | — |  |  |  | |  |  |
| 5. Parental education | -.120^*^ | .141^**^ | .345^**^ | -.038 | — |  |  | |  |  |
| 6. Household Income | -.083 | .073 | .207^**^ | -.035 | .399^**^ | — |  | |  |  |
| 7. Adolescent depressed mood | .316^**^ | -.234^**^ | -.155^**^ | .162^**^ | -.126^**^ | -.176^**^ | — | |  |  |
| 8. Parental closeness | -.222^**^ | .147^**^ | .089 | -.110^**^ | .093^**^ | .130^**^ | -.372^**^ | | — |  |
| 9. Peer acceptance | -.194^**^ | .181^**^ | .140^**^ | -.090^**^ | .094^**^ | .134^**^ | -.306^**^ | | .389^**^ | — |

^*^*p* < .05. ^**^*p* < .01. ^***^*p* < .001.
