# Supplementary material for: Tracking of depressed mood from adolescence into adulthood and the role of peer and parental support: A partial test of the Adolescent Pathway Model
Source: SSM Popul Health. 2023 May 26;23:101440. doi: 10.1016/j.ssmph.2023.101440 (PMC10492161; doi:10.1016/j.ssmph.2023.101440)
Supplement: Multimedia component 4 [file mmc4.docx]

**Appendix D. Attrition analysis**

To test for attrition patterns, we grouped respondents into non-respondents and respondents (coded as 0 and 1). We conducted independent sample t-tests to see if there were any differences in parental education, household income, peer acceptance and parental closeness among respondents who stayed and dropped out of the study by 2017. The independent sample t-tests showed that respondents who stayed in the study until 2017 had significantly higher levels of parental education and household income (See Table D1). No differences were found for peer acceptance and parental closeness. An earlier study on the same dataset further revealed that no differences were found in adolescent depressed mood for those who participated versus dropped out at age 40 (Jørgensen, 2023). This study also showed a small positive association between gender and attrition through chi-square testing. Based on proportions only, females were slightly more likely to participate at the last time point.

**Table D1.** Independent sample t-tests for responders and non-responders at age 40 (2017)

| Participation | Yes |  | No | |  |  | |  | |  |  | |  | |  |
| --- | --- | --- | --- | --- | --- | --- | --- | --- | --- | --- | --- | --- | --- | --- | --- |
|  | *n* | Mean  (SD) | *n* | Mean  (SD) | Mean difference | | F-value | | Two-tailed p-value | | | t- value |  | df | |
| Parental education | 419 | 2.84  (1.19) | 508 | 2.57  (1.09) | -.28 | | 8.31 | | P < .001 | |  | -3.67 | | 925 | |
| Household income | 322 | 4.45  (2.00) | 293 | 4.12  (1.26) | -.33 | | .72 | | .001 | |  | -3.30 | | 613 | |
| Parental closeness | 448 | 4.43  (.86) | 617 | 4.46  (.90) | .03 | | 1.03 | | .61 | |  | .51 | | 1063 | |
| Peer acceptance | 450 | 4.63  (.77) | 633 | 4.66  (.81) | .03 | | .46 | | .58 | |  | .56 | | 1081 | |

**References**

Jørgensen, M., Smith, O.R.F., Wold, B. & Haug, E. (2023). *Associations between life transitions into adulthood and depressed mood over time: A 27-year longitudinal study focused on adolescent socioeconomic determinants*. [Manuscript submitted for publication]. Department of Health Promotion and Development, University of Bergen.
